# A G protein‐coupled α7 nicotinic receptor regulates signaling and TNF‐α release in microglia

**DOI:** 10.1002/2211-5463.12270

**Published:** 2017-08-07

**Authors:** Justin R. King, Trudy C. Gillevet, Nadine Kabbani

**Affiliations:** ^1^ Interdisciplinary Program in Neuroscience Krasnow Institute for Advanced Study George Mason University Fairfax VA USA; ^2^ School of Systems Biology Krasnow Institute for Advanced Study George Mason University Fairfax VA USA

**Keywords:** acetylcholine, GTP‐binding protein, immune cells, inflammation, lipopolysaccharide

## Abstract

Acetylcholine activation of α7 nicotinic acetylcholine receptors (α7 nAChRs) in microglia attenuates neuroinflammation and regulates TNF‐α release. We used lipopolysaccharide to model inflammation in the microglial cell line EOC20 and examined signaling by the α7 nAChR. Co‐immunoprecipitation experiments confirm that α7 nAChRs bind heterotrimeric G proteins in EOC20 cells. Interaction with Gαi mediates α7 nAChR signaling via enhanced intracellular calcium release and a decrease in cAMP, p38 phosphorylation, and TNF‐α release. These α7 nAChR effects were blocked by the inhibition of Gαi signaling via pertussis toxin, PLC activity with U73122, and α7 nAChR channel activity with the selective antagonist α‐bungarotoxin. Moreover, α7 nAChR signaling in EOC20 cells was significantly diminished by the expression of a dominant‐negative α7 nAChR, α7_345‐8A,_ shown to be impaired in G protein binding. These findings indicate an essential role for G protein coupling in α7 nAChR function in microglia leading to the regulation of inflammation in the nervous system.

**Abbreviations**
ACh: acetylcholine
BGTX: α‐bungarotoxin
cAMP: cyclic adenosine monophosphate
Chol: choline
CNS: central nervous system
Co‐IP: co‐immunoprecipitation
fBGTX: fluorescent α‐bungarotoxin
G Protein: heterotrimeric G protein
GPBC: G protein‐binding cluster
GPCR: G protein‐coupled receptor
Gα: heterotrimeric G protein Subunit α
IP_3_: inositol triphosphate
IP_3_R: inositol triphosphate receptor
IP: immunoprecipitation
LPS: lipopolysaccharide
MEC: mecamylamine
NO: nitric oxide
p38: p38 mitogen‐activated protein kinase
PC12: pheochromocytoma Cell line 12
phospho‐p38: phosphorylated p38 mitogen‐activated protein kinase
phosphor‐CDC42: Cell Division Control protein 42
PLC: phospholipase C
PTX: pertussis toxin
ROI: region of interest
ROS: reactive oxygen species
TNF‐α: tumor necrosis factor α
Xest. C: xestospongin C
α7 nAChR: α7 Nicotinic Acetylcholine Receptor

Microglia are the primary immune cells of the central nervous system (CNS), and during disease such as stroke and HIV infection, they are activated to promote widespread CNS inflammation 1, 2. Activated microglia secrete cytokines such as tumor necrosis factor α (TNF‐α) and interleukin‐1b, which drive neuroinflammatory signaling in neurons and non‐neuronal cells 3. Microglia also exert important neuroprotective function by secreting growth and anti‐inflammatory factors 4. Acetylcholine (ACh) potently regulates immune cells such as macrophages and microglia 5. Interestingly, cholinergic neurons are also susceptible to neuroinflammatory insults, which can underlie the pathology of disorders such as Alzheimer's disease (AD) and neurocognitive degeneration associated with brain HIV infection 6, 7. The pharmacological targeting of α7 nAChRs is thus a promising approach for the treatment of neuroinflammation 7, 8, 9.

Receptors that bind ACh are divided into two main categories: (a) ionotropic nicotinic receptors (nAChRs), which are fast‐responding cation channels, and (b) metabotropic muscarinic receptors, which are slower‐responding G protein‐coupled receptors (GPCRs) 10, 11. Both types of receptors are expressed in microglia but α7 nAChRs play a vital role in inhibiting the release of inflammatory factors such as TNF‐α, and nitric oxide (NO), and reactive oxygen species (ROS) 12, 13. Studies show that ACh, as well as nicotine, can negatively regulate the release of inflammatory cytokines through α7 nAChR signaling via phospholipase C (PLC), intracellular calcium, and the phosphorylation of p44/42 and p38 mitogen‐activated protein kinase (p38) 5, 14.

More specifically, α7 nAChRs are homopentameric channels that operate via both ion flux and the ability to increase intracellular calcium from the ER in various types of cells 15. It is also likely that ligand‐bound α7 nAChRs operate through metabotropic signaling in nonexcitable cells such as immune cells 16. Recent work from our laboratory indicates that α7 nAChRs bind and activate heterotrimeric G proteins such as Gαq leading to cytoskeletal remodeling and growth in differentiating pheochromocytoma 12 (PC12) cells 17, 18, 19, 20. In T‐cell lymphocytes, α4β2 nAChRs signal via Gαi to mediate the release of Th1‐type cytokines in response to nicotine 21. In this study, we explore the role of α7 nAChR/G protein interactions in microglia. Our results indicate that signaling through Gαi underlies an ability of the α7 nAChR to regulate intracellular calcium, p38 activity, and TNF‐α release from microglial cells.

## Materials and methods

### Cell culture, transfection, and protein extraction

EOC20 cells (ATCC^®^ CRL‐2469, Manassas, VA, USA) were grown on plastic petri dishes or glass coverslips (Genesee Scientific, San Diego, CA, USA) coated with a poly‐d‐lysine (100 μg·mL^−1^) matrix and maintained in DMEM (Thermo Fisher, Waltham, MA, USA) supplemented with 10% fetal bovine serum and 1% penicillin/streptomycin (Thermo Fisher). Mouse macrophage colony‐stimulating factor 1 (M‐CSF1) was added to the culture media as specified by ATCC (Pro Spec Bio, East Brunswick NJ, USA). 1 μg·mL^−1^ Lipopolysaccharide (LPS) treatment for 4 h or more was used to promote inflammatory responses in EOC20 cells 5.

Cells were transfected with cDNA plasmids for α7_345‐348A_ (pcDNA 3.1), which is impaired in G protein coupling and functions as a dominant negative for G protein signaling 17, and GCaMP5G 22 using Lipofectamine 2000 (Thermo Fisher). Transfection with the corresponding empty vector was used as a transfection control in the experiments. Plasmid DNA was purified by maxi prep (Zymo Research, Irvine, CA, USA). For protein analysis, cultured cells were detached using trypsin and then lysed with a nondenaturing lysis solution consisting of 1% Triton X‐100, 137 mm NaCl, 2 mm EDTA, 20 mm Tris/HCl (pH 8), and a protease/phosphatase inhibitor cocktail (Roche, Penzberg, Germany). For western blot or co‐immunoprecipitation (co‐IP) experiments, proteins were prepared from cellular membrane fractions as described 19. Protein concentration was determined using the Bradford protein assay kit (Thermo Fisher).

### Drugs

α7 nAChR‐specific agonist choline (10 mm, 3 mm, and 1 mm) (Acros Organics, Geel, Belgium) 17; IP_3_R antagonist xestospongin C (Xest. C) (1 μm) (Tocris, Bristol, UK) 18; Gαi signaling blocker pertussis toxin (Ptx) (100 ng·mL^−1^) (Calbiochem, San Diego, CA, USA)^23^; α7 nAChR antagonists α‐BGTX (50 nm) 18, 24 and mecamylamine (10 μm) 25, 26; phospholipase C inhibitor U73122 (10 μm) 27; and the adenylate cyclase activator forskolin (10 μm) were used for this study 28.

### Immunoprecipitation and western blot

Immunoprecipitation (IP) or co‐IP of the α7 nAChR was performed as described 19. Briefly, a co‐IP of the α7 nAChR protein complex was obtained from 500 μg cell membrane protein fraction using 5 μg of the C‐20 antibody (Santa Cruz, Dallas, TX, USA) 19. Protein complexes associated with the co‐IP were captured using a Protein G Dynabeads (Thermo Fisher). For western blot detection, 100 μg of protein was loaded into each lane of an SDS/PAGE gel. Proteins were transferred onto a nitrocellulose membrane (Thermo Fisher) for immunoblot detection using the following antibodies: anti‐Gαs (Rabbit) (New East Bioscience), anti‐Gαq (Rabbit) (New East Bioscience), anti‐Gαi (Rabbit) (New East Bioscience), anti‐Gβ (T‐20) (Santa Cruz), anti‐phospho‐p38MAPK (Thr180/Tyr182), anti‐p38MAPK, anti‐phospho‐CDC42/Rac1 (Ser 71), and anti‐GAPDH (Cell Signaling, Danvers, MA, USA). Horseradish peroxidase (HRP)‐conjugated secondary antibodies were purchased from Jackson ImmunoResearch (West Grove, PA, USA). Immunoblots were visualized using SuperSignal West Pico Chemiluminescence (Thermo Fisher) via a G:Box imaging system and GeneSYS software (Syngene, Fredrick MD, USA). SeeBlue protein standard (Thermo Fisher) was used as a molecular weight marker.

### Calcium imaging

EOC20 cells were transfected with the calcium sensor protein GCaMP5G 22 3 days prior to the calcium imaging experiment. Changes in intracellular calcium were measured using an inverted Zeiss LSM800 confocal microscope at an acquisition rate of 1 frame per 256 ms for 75 s at 2 × 2 binning. Phototoxicity and bleaching were minimized using low‐wavelength and neutral density light filters 29. Choline (1–10 mm) was directly applied to the recording chamber after the capture of a 50‐frame baseline signal. Calcium transients were measured as ΔF/F_θ_ using ImageJ (NIH).

### cAMP imaging

EOC20 cells were transduced with a viral vector for imaging active cyclic adenosine monophosphate (cAMP) (Montana Molecular, Bozeman, MT, USA). Cell transduction was performed in accordance with the manufacturer's protocol for the red fluorescent cADDis cAMP assay 48 h before imaging. Whole cells were imaged during treatment with 3 mm choline for 4 min or 3 mm choline for 4 min after a 30‐min pretreatment with either the Gαi inhibitor PTX or the α7 nAChR inhibitor BGTX. Cells were treated with 10 μm forskolin as a positive control in the cAMP assay. Images were captured every minute following a 30‐s baseline recording. Imaging was performed using an inverted Zeiss LSM800 confocal microscope, and fluorescence signal was analyzed using ImageJ.

### Enzyme‐linked immunosorbent assay

Tumor necrosis factor α release was stimulated using 1 μg·mL^−1^ lipopolysaccharide (LPS) stimulation of EOC20 cells. In some experiments, cells were pretreated with PTX/Xest C. for 1 h or choline (1 mm) for 30 min prior to the application of LPS as indicated in the text. Extracellular TNF‐α was measured from the cell culture medium using an ELISA kit (R & D systems) 5. Experiments were performed in triplicate.

### Statistics

Group averages were obtained for all experiments, and each assay was run in triplicate. Data were analyzed via one‐way and two‐way ANOVA, or Student's t‐test where appropriate, using the SPSS 24 statistics package to determine significance between mean values. Fischer's LSD post hoc tests were used for all individual comparisons where appropriate. A minimum statistical value *P* < 0.05 was considered significant.

## Results

### α7 nAChRs bind heterotrimeric G proteins in microglial cells

**Figure 1:**
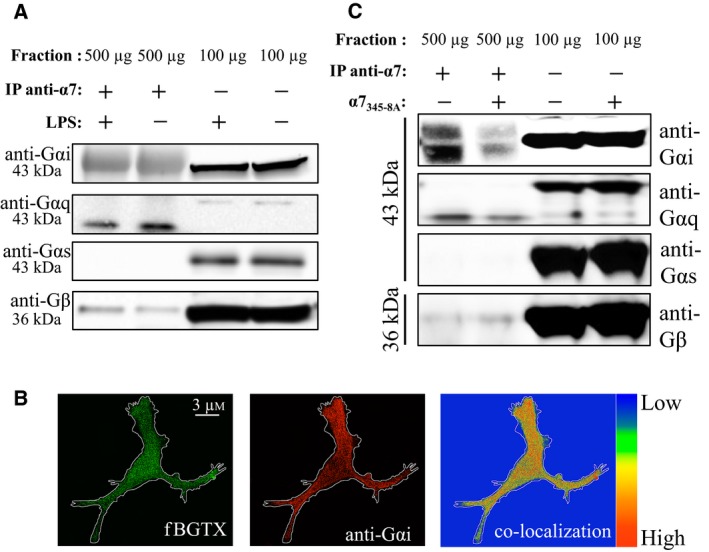
α7 nAChRs interact with G proteins in microglial cells. (A) Co‐immunoprecipitation (co‐IP) of the α7 nAChR from EOC20 cells using the anti‐C20 antibody. Western blot detection using anti‐Gαi, anti‐Gαq, anti‐Gαs, and anti‐Gβγ antibodies. Cells were treated with LPS (1 μg·mL^−1^) for 4 h prior to the co‐IP. (B) Double labeling of EOC20 cells with fBGTX (at the cell surface) and anti‐Gαi antibodies. A heat map shows co‐expression of the two proteins. (C) Co‐IP of the α7 nAChR from cells transfected with the dominant‐negative α7_345‐8A_ or an empty vector. 100 μg of total membrane fraction was used as a positive control (*n* = 3).

Studies have demonstrated a role for α7 nAChRs in the modulation of inflammatory signaling in microglial cells 5, 14. α7 nAChRs have been shown to signal through intracellular proteins such as G proteins in primary microglia, in several cell lines, and in native tissue 17, 19. We have observed a functional role for direct interactions between α4 subunit containing nAChRs and G proteins in T cells 21. To test for interaction between α7 nAChRs and G proteins in microglia, we used an α7 nAChR co‐immunoprecipitation (co‐IP) approach based on the ability of the anti‐α7 nAChR C‐20 antibody to isolate receptor–protein complexes from cultured cells 17, 18. Interaction between α7 nAChRs and various G protein subunits was surveyed in the co‐IP assay using western blot. The same α7 nAChR co‐IP experiment was performed in membrane fractions of 4‐h LPS‐activated EOC20 cells. EOC20 cells express endogenous α7 nAChRs and have been used as a microglial model system to study the function of this receptor 30. As shown in Fig. 1A, co‐IP experiments confirm interaction between endogenous α7 nAChRs and G proteins in EOC20 cells. Specifically, we detected a strong anti‐Gαi‐immunoreactive band and a fainter anti‐Gβγ band within the α7 nAChR co‐IP (Fig. 1A). An anti‐Gαq‐immunoreactive band was also visualized at 43 kDA within the co‐IP but this band did not match the molecular weight of the anti‐Gαq‐immunoreactive band observed in the total membrane fraction, which ran at a higher molecular weight on the gel (Fig. 1A). LPS treatment did not appear to alter the interaction between the G proteins and the α7 nAChR in the co‐IP experiment. Anti‐Gαs immunoreactivity was not observed in the α7 nAChR co‐IP experiment.

To confirm expression of cell surface α7 nAChRs, we labeled fixed EOC20 cells with a fluorescent‐conjugated BGTX (fBGTX) prior to plasma membrane permeabilization with triton. As shown in Fig. 1B, fBGTX was detected on the cell surface of EOC20 cells consistent with evidence on the expression of α7 nAChRs at the plasma membrane of microglia 5. Double labeling of EOC20 cells with an anti‐Gαi antibody and fBGTX indicates colocalization of Gαi and α7 nAChRs in microglial cells (Fig. 1B). The data suggest that α7 nAChRs associate with Gαi proteins in microglial cells, consistent with earlier findings on the interaction between nAChRs and Gαi in T cells 21.

### The G protein‐binding cluster contributes to α7 nAChR/G protein association

An intracellular M3‐M4 loop ranging from 90 to 110 amino acids exists in nAChRs serving as a domain for receptor–protein interactions 31, 32, 33. Recently, we have shown that four amino acids within the intracellular M3‐M4 loop of the α7 nAChR are crucial for G protein binding 17. This site is termed the G protein‐binding cluster (GPBC) and is conserved in the structurally related glycine receptor 34. We created and functionally characterized a dominant‐negative α7 nAChR subunit that is deficient in G protein binding based on a mutation of the GPBC (α7_345‐8A_) 17. Here, we utilized α7_345‐8A_ to test the role of the GPBC in α7 nAChR/G protein interaction in microglial cells. EOC20 cells were transiently transfected with plasmids encoding α7_345‐8A_ 72 h prior to the co‐IP experiment. As shown in Fig. 1C, expression of α7_345‐8A_ was sufficient to occlude much of the association between the α7 nAChR and Gαi. In contrast, little to no change in Gβγ binding was observed in cells transfected with α7_345‐8A_ relative to the empty vector‐transfected controls (Fig. 1C). A reduction in the anti‐Gαq‐reactive band was also observed in the co‐IP in cells transfected with α7_345‐8A_ and we did not detect an anti‐Gαs signal on the blot (Fig. 1C). The results indicate that the GPBC is necessary for interaction between α7 nAChRs and G proteins in EOC20 cells.

### Choline activation of α7 nAChRs promotes intracellular calcium transients in microglial cells in a G protein‐ and dose‐dependent manner

**Figure 2:**
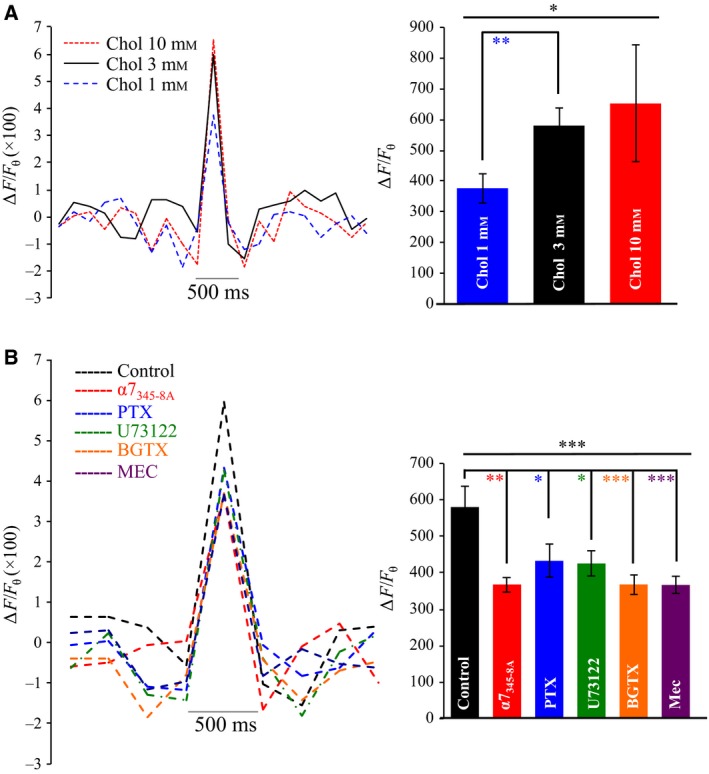
α7 nAChRs regulate intracellular calcium release in EOC20 cells. (A) Left, calcium transient responses (Δ*F*/*F*_θ_) measured by GCaMP5G fluorescence (Chol: 1 mm, 3 mm, and 10 mm). Corresponding histogram shows the average of the peak calcium value across groups. (B) Intracellular calcium transient responses following treatment with 3 mm Chol alone (black) or 3 mm Chol in cells transfected with α7_345‐8A_ expression (red); preincubated with 100 ng·mL^−1^ pertussis toxin (PTX) (blue line); 10 μm U73122 (green); 50 nm bungarotoxin (BGTX) (orange); 10 μm mecamylamine (Mec) (purple). Corresponding histogram shows the average of the peak calcium value across groups. (***P < 0.001, **P < 0.01, *P < 0.05, *n* = 9+ per group)

Activation of α7 nAChRs increases intracellular calcium levels in neurons, astrocytes, and non‐neural cells through both ionotropic and ER store‐mediated calcium release 17, 18, 19, 35. We have shown that α7 nAChR stimulation leads to calcium release from the ER through the activation of Gαq and PLC in neural cells 17, 18. A similar pathway in primary microglia enables α7 nAChR‐mediated neuroprotection following P2X_7_ stimulation 14. We tested the ability of α7 nAChRs to promote intracellular calcium release from the ER in EOC20 cells. Real‐time calcium fluctuations were visualized by genetic expression of the calcium sensor protein GCaMP5G. α7 nAChRs were pharmacologically activated with the specific agonist choline at concentrations associated with full receptor occupancy (1 mm, 3 mm, and 10 mm; EC50:≈0.9–1.5 mm 36, 37). In response to choline, a rapid intracellular calcium transient was observed in EOC20 cells [ANOVA: *F*(2,35) = 16.659; *P* = 0.004]. At 1 mm, choline increased intracellular calcium levels by 376% (±47.92) from the baseline. At 3 mm, choline increased intracellular calcium levels to 580% (±56.46) (post hoc comparison *P* = 0.002; compared to 1 mm Chol treatment) of the baseline. At the highest dose of 10 mm, choline increased intracellular calcium levels to 652% (±189.14) at the peak of the transient consistent with a dose‐dependent saturation of the receptor‐binding site. An analysis of the duration of the calcium transient response to choline indicates that the average calcium transient lasts for approximately 500mSec at all tested concentrations (Fig. 2A).

A combination of pharmacology and genetic mutation was used to demonstrate a role for Gαi and PLC in α7 nAChR‐mediated calcium transient responses in EOC20 cells. This was evidenced by a significance difference in the peak calcium transient responses among various treatment groups [ANOVA: *F*(5,79) = 4.877; *P* = 0.001]. Specifically, expression of the α7_345‐8A_ nAChR mutant significantly attenuated the ability of 3 mm choline to foster an intracellular calcium response relative to the empty plasmid transfection control group (α7_345‐8A_ peak = 367.67±20.4% vs. control peak = 580.35% ± 56.4%; post hoc comparison *P* = 0.005). Expression of α7_345‐8A_ did not change the duration of the calcium transient relative to controls (Fig. 2B). To examine the role of Gαi in choline‐mediated intracellular calcium transient responses, cells were pretreated with the selective Gαi blocker pertussis toxin (PTX) (100 ng·μL^−1^) for 30 min. In this condition, we observed a significant reduction in the peak of the intracellular calcium response to choline (α7 + PTX peak = 432.88 ± 44.6%; post hoc comparison to control *P* = 0.027) relative to the choline treatment control. Inhibition of PLC activity by pretreatment U73122 (10 μm) prior to 3 mm choline stimulation was associated with a significant reduction in the peak of the calcium transient relative to the choline treatment alone (U73122 peak = 425.57 ± 34.4%) (post hoc comparison *P* = 0.030 to Chol 3 mm alone). Preincubation of cells with the α7 nAChR‐specific antagonist BGTX (50 nm) or the broad nAChR antagonist mecamylamine (Mec) (10 μm) was associated with a loss in the calcium transient response to choline (BGTX = 368.03% ± 27.36, post hoc comparison *P* = 0.001 to Chol 3 mm alone; Mec = 366.67% ± 23.29, post hoc comparison *P* = 0.001 to Chol 3 mm alone).

### α7 nAChR regulation of p38 depends on Gαi activity

**Figure 3:**
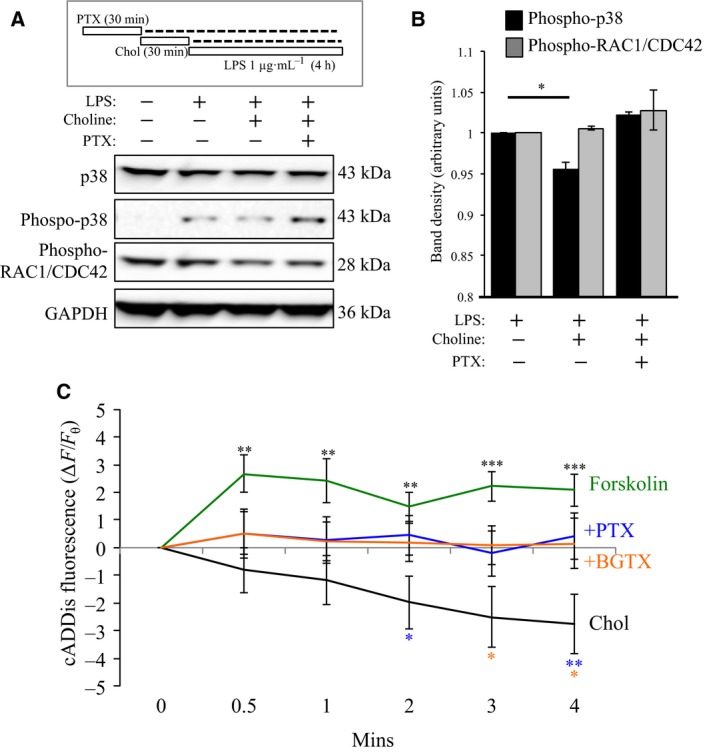
α7 nAChR/G protein interaction regulates p38 phosphorylation and cAMP levels. (A) Gantt chart of the experiment: Chol (1 mm); PTX (100 ng·mL^−1^); LPS (1 μg·mL^−1^). Western blot detection of total p38, phospho‐p38, and phospho‐RAC1/CDC42 expression in EOC20 cells. Anti‐GAPDH detection was used to confirm loading across lanes. (B) Average values of the band density for three separate experiments (*n* = 3) in (A) demonstrate a significant effect of choline on phospho‐p38 level. (C) cAMP detection in EOC20 cells using real‐time cADDis fluorescence measure. Cells were treated with 3 mm Chol (black) or 3 mm Chol following pretreatment with 100 ng·mL^−1^ PTX (blue); 50 nm BGTX (orange). Treatment with 10 μm forskolin (green) was used as a positive control in the cAMP assay. (***P < 0.001, **P < 0.01, *P < 0.05, *n* = 8+ per group)

Members of the MAPK family (ERK, JNK, and p38) are involved in LPS‐induced TNF‐α production and the inflammatory response 5, 38, 39. A variety of cellular stressors, including LPS, activate p38 MAPK (p38) by phosphorylation at Thr180 and Tyr182 40. Nicotine has been found to inhibit LPS‐induced phosphorylation of JNK, p38, and interfere with post‐transcriptional regulation of TNF‐α in microglia 5. We tested the ability of α7 nAChRs to regulate p38 expression and phosphorylation at Thr180/182 using an antibody selective for phosphorylation at this site (phospho‐p38). LPS treatment was associated with an increase in phospho‐p38 levels (Fig. 3A,B). Treatment with choline (1 mm; 30 min) significantly attenuated the intensity of the phospho‐p38 band signal [ANOVA: *F*(2,8) = 37.508; *P* < 0.001]; choline vs. control *P* = 0.001) (Fig. 3A,B). In cells pretreated with PTX (100 ng·μL^−1^; 30 min), choline did not attenuate phospho‐p38 levels from the LPS baseline, suggesting that Gαi activity is required for α7 nAChR‐mediated regulation of p38 in the cell. Here, differences in phospho‐p38 expression appeared due to specific phosphorylation at Thr180/182 residues as overall levels of p38 expression remained unchanged across the experimental conditions (Fig. 3A,B).

We have shown a role for the GPBC in mediating α7nAChR regulation of the actin cytoskeleton through the activation of Rho family GTPases 20. In immune cells, the Rho family GTPases RAC1 and CDC42 can regulate inflammatory signaling upstream of nuclear factor‐κB (NF‐κB) 41. We examined the ability of the specific α7 nAChR ligand choline to regulate RAC1/CDC42 in EOC20 cells. Using an antibody that recognizes an AKT‐specific phosphorylation site (Ser 71) involved in GTP binding in RAC1 and CDC42 42, we determined the effect of α7 nAChR activity on these Rho family proteins. As shown in Fig. 3A,B, choline had little effect on phospho‐RAC1/CDC42 expression relative to the LPS control group. In these experiments, pretreatment with PTX (100 ng·mL^−1^; 30 min) had no effect on phospho‐RAC1/CDC42 band density, suggesting that these Rho GTPases are not regulated by α7 nAChR/Gαi interaction.

### α7 nAChR activation of Gαi attenuates cAMP levels in EOC20 cells

Cyclic adenosine monophosphate (cAMP) is an important second messenger in pathways underlying inflammatory responses in microglia and macrophages 43, 44, 45. Studies in primary microglia indicate that cAMP production by Gαs regulates the release of TNF‐α 44. We tested the role of α7 nAChR/Gαi coupling in cAMP production in EOC20 cells using the newly developed fluorescent cAMP sensor cADDis. Treatment of cells with the adenylate cyclase activating compound forskolin (10 μm) 28 was associated with a rapid significant rise in cADDis fluorescence that peaked at 4 min after drug application (+2.078 ± 0.508), consistent with rapid cAMP production in the cell. Treatment with 3 mm choline, on the other hand, was associated with a significant decrease in cADDis fluorescence at that time (−2.764±1.065), suggesting that activation of the α7 nAChR leads to a decrease in cellular cAMP levels. This effect of choline on cAMP was inhibited by pretreatment of cells with PTX (100 ng·mL^−1^) or BGTX (50 nm). An ANOVA was ran at each time point to look for significance between groups, with Fisher's LSD test used to compare groups. Significant differences were found at all time points: 0.5 min [ANOVA: *F*(3,49) = 27.746; *P* = 0.035]; 1 min [ANOVA: *F*(3,49) = 29.451; *P* = 0.034]; 2 min [ANOVA: *F*(3,49) = 30.405; *P* = 0.019]; 3 min [ANOVA: *F*(3,49) = 51.865; *P* = 0.004]; 4 min [ANOVA: *F*(3,49) = 58.049; *P* = 0.003]. When looking at post hoc analysis, forskolin leads to significant increases at all time points when compared with choline treatment (0.5 min *P* = 0.004; 1 min *P* = 0.004; 2 min *P* = 0.003; 3 min *P* < 0.001; 4 min *P* < 0.001), while treatment with the antagonists PTX and BGTX both significantly attenuated changes in cAMP caused by choline following 2, 3, and 4 min of activity, respectively. These findings support a role for Gαi inhibition of the cAMP pathway in α7 nAChR signaling in microglial cells.

### α7 nAChR inhibits TNF‐α release through G protein‐coupled ER calcium release

**Figure 4:**
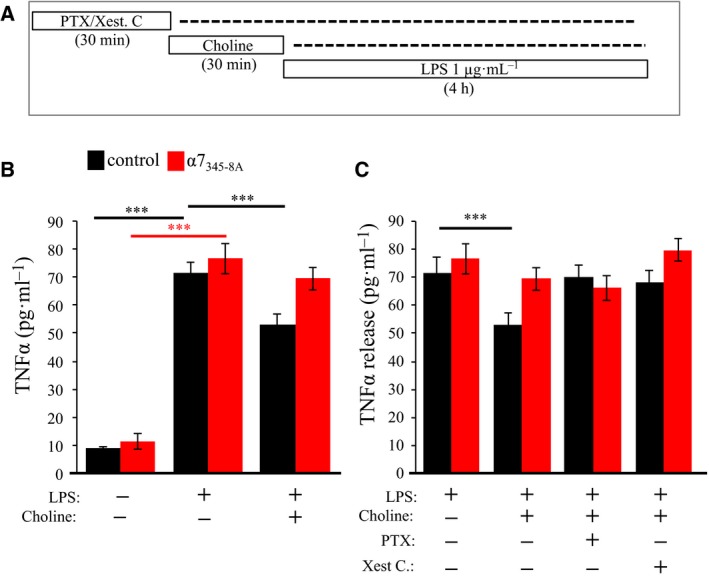
α7 nAChR regulation of TNF‐α release is Gαi and IP_3_ receptor sensitive. (A) Gantt chart of the ELISA experiment: Chol (1 mm); PTX (100 ng·mL^−1^); Xest. C. (1 μm); LPS (1 μg·mL^−1^). (B,C) TNF‐α levels were measured in the extracellular media following drug treatment. Black: transfection control group that was transfected with an empty vector; red: cells transfected with α7_345‐8A_. (***P < 0.001, *n* = 8+)

Hallmarks of microglial activation are cell proliferation, nitric oxide (NO), TNF‐α, and reactive oxygen species (ROS) production and release 46. ACh has been shown to be anti‐inflammatory in both the CNS and periphery and can directly attenuate the release of TNF‐α from immune cells 5, 14. We examined the role of α7 nAChR/G protein signaling on TNF‐α release in EOC20 cells. Total levels of TNF‐α released from cultured cells following LPS activation was measured using an ELISA method (Diagram Fig. 4A). As shown in Fig. 4B and Table 1, a 4‐h exposure of LPS was found to significantly increase the amount of TNF‐α released by sevenfold (*P* < 0.001) compared to non‐LPS‐stimulated cells. In cells transfected with α7_345‐8A,_ LPS treatment was found to significantly increase the amount of TNF‐α released to a similar extent as the native (nonmutated) receptor‐expressing cells (*P* < 0.001) (Fig. 4B and Table 1). No change in baseline TNF‐α release was observed in cells transfected with α7_345‐8A_ in the absence of LPS.

**Table 1:** Tumor necrosis factor α release from EOC20 cells is regulated by α7 nAChR/G protein signaling. An ELISA was used for the quantification of TNF‐α released from EOC20 cells under various experimental conditions. Cells transfected with an empty vector were used as controls for cells transfected with α7_345‐8A_. Statistically significant values (*P* < 0.05) are indicated in boldface

Treatment	Control			α7_345‐8A_		
	TNF‐α levels (pg·mL^−1^)	±SEM	% Change from Baseline with LPS	TNF‐α levels (pg·mL^−1^)	±SEM	% Change from Baseline with LPS
Baseline (no LPS)	8.79	0.67	–	11.41	2.82	–
Baseline (with LPS)	**71.75**	**3.38**	–	**76.56**	**5.35**	–
LPS + Chol 1 mm	**53.27**	**3.43**	−**25.75%**	69.50	4.08	−9.22%
LPS + Chol 1 mm (PTX 100 ng·mL^−1^)	70.08	3.35	−2.32%	66.18	4.40	−13.56%
LPS + Chol 1 mm (Xest C. 1 μm)	68.38	4.58	−4.70%	79.77	4.13	4.20%

We tested the effect of choline on TNF‐α release in LPS‐activated microglia. As shown in Fig. 4C and Table 1, choline was found to significantly attenuate the release of TNF‐α in control cells transfected with an empty vector [ANOVA: *F*(2,117) = 158.77 *P* < 0.001]. Transfection with α7_345‐8A_ was associated with a loss in the effect of choline on TNF‐α release resulting in extracellular TNF‐α levels comparable to the noncholine‐treated cohort. We confirmed the involvement of Gαi in α7 nAChR‐mediated inhibition of TNF‐α release. As shown in Fig. 4C and Table 1, pretreatment with PTX was found to inhibit the effect of choline on TNF‐α release. Pretreatment with PTX had no effect on extracellular TNF‐α levels in α7_345‐8A_‐transfected cells [ANOVA: *F*(4,145) = 97.757; *P* < 0.001] relative to the LPS baseline (Fig. 4B,C and Table 1). The findings suggest that Gαi activity is needed for α7 nAChR inhibition of TNF‐α release. A two‐way ANOVA testing for interaction between receptor type and drug treatment revealed a nonsignificant interaction between the two variables (*P* = 0.102); however, a significant interaction was established between the LPS treatment alone and the LPS treatment with choline condition (*P* < 0.001).

Studies indicate that nicotine modulates cellular calcium levels by stimulating release from the ER 47. α7 nAChR‐mediated activation of IP_3_ receptors in neurons and microglia promotes intracellular calcium signaling through the activity of the ER 14, 17. We tested the role of IP_3_ receptors in choline‐associated TNF‐α release. LPS‐activated EOC20 cells were pretreated with the IP_3_ receptor antagonist xestospongin C (Xest. C) (1 μm; 30 min) prior to choline stimulation and ELISA analysis 48 (Fig. 4C). In Xest. C‐pretreated cells, choline did not attenuate TNF‐α release when compared to the LPS baseline condition (Fig. 4C and Table 1). The data show that IP_3_ receptor activation is necessary for α7 nAChR regulation of TNF‐α release from microglial cells. In cells transfected with α7_345‐8A_, pretreatment with Xest. C was also found to suppress the effect of choline on TNF‐α release (Fig. 4C). These findings indicate that α7 nAChRs modulate TNF‐α release through both Gαi activity and intracellular calcium release through the IP_3_ receptor.

## Discussion

Activation of α7 nAChRs in immune cells promotes anti‐inflammatory signaling, which can be of pharmacological value for the treatment of disorders such as asthma, ulcerative colitis, and arthritis 49. Anti‐inflammatory signaling through α7 nAChRs may also be useful for the development of new therapies for neurodegenerative disorders such as Alzheimer's and Parkinson's disease 50, 51. A better understanding of how α7 nAChRs operate in immune cells is essential for drug development, and our results indicate that α7 nAChRs operate through a G protein signaling pathway in microglial cells. These findings are consistent with our earlier studies that demonstrate an ability of nAChRs to function through G proteins in neural and immune cells 52. α7 nAChR/G protein interactions thus offer a new molecular target in drug development for the treatment of brain disease.

Our findings indicate that α7 nAChRs activate G protein‐associated pathways in EOC20 cells in response to ligand stimulation, suggesting that nAChRs operate in a metabotropic manner in microglia; however, this hypothesis remains to be fully tested as transactivation of GPCRs cannot be excluded from our results. Recent findings on glutamate binding kainate receptors (KAR) show that this class of ion channels can also activate Gαo signaling via a direct interaction between the channel and the G protein 53. Because the prokaryotic homolog of the nAChR, GLIC, does not contain an intracellular (M3‐M4 loop) protein‐binding domain for cell signaling 54, it is tempting to speculate that the ability of nAChRs to engage G protein signaling emerged later as an auxiliary‐to‐ionotropic function. The precise molecular mechanism that allows ion channel proteins to activate Gα subunits is an important direction for future study. The current findings provide compelling evidence on the role of α7 nAChR/G protein interactions in microglial cells. Future studies on the role of α7 nAChR/G protein signaling in primary microglia and *in vivo* are now required to confirm and delineate the role of G protein signaling in nAChR‐mediated regulation of inflammation.

Our findings indicate that α7 nAChR activation of the Gαi pathway promotes an increase in intracellular calcium through IP_3_ receptors on nearby ER. Related findings in neurons indicate that α7 nAChRs localize near the ER and function to regulate intracellular calcium signaling 18, 55. Ligand binding to the α7 nAChR stimulates PLC activity in PC12 cells and primary microglia 14, 17. In this study, interaction of the α7 nAChR with Gαi was found to promote IP_3_‐mediated ER calcium release, resulting in the inhibition of TNF‐α from EOC20 cells. Our data imply the presence of an anti‐inflammatory α7 nAChR/Gαi pathway in microglial cells that may operate through PLC activity. This pathway is supported by four key lines of evidence: (a) Intracellular calcium transient measures in EOC20 cells are found to be dramatically diminished in cells transfected with the mutant receptor α7_345‐8A_, which strongly weakens the interaction between the endogenous α7 nAChR and the G protein; (b) pretreatment of cells with the Gαi blocker PTX attenuates the choline‐mediated calcium transient response in microglial cells to the same extent as interference with the α7/G protein interaction with α7_345‐8A_; (c) Gαi and Gβγ subunits can each activate PLC in cells and inhibition of PLC with the U73122 leads to a decrease in intracellular calcium transients similar to those seen when blocking with PTX, or following α7_345‐8A_ expression 56, making this enzyme a highly likely downstream target; (d) TNF‐α release from microglia is regulated by the α7 nAChR/Gαi pathway as demonstrated by the functional effects of PTX, α7_345‐8A_ expression, and IP_3_ receptor antagonism with Xest C.

The anti‐inflammatory actions of the α7 nAChR in microglia appear to be regulated at the level of transcriptional control of molecules such as NF‐κB and p38 kinase 5, 13. In primary microglia, nicotine has been shown to inhibit LPS‐induced activation of JNK and p38 14. Our experiments in EOC20 cells indicate that choline activation of the α7 nAChR can also attenuate the phosphorylation of p38 in a PTX‐sensitive manner, confirming the role of Gαi in this process. This pathway appears to be specific to targets such as p38 as we did not detect an effect of choline on the phosphorylation of RAC1/CDC42 in the same cell. In addition, our study suggests a role for α7 nAChR‐mediated Gαi inhibition of cAMP production in the inhibition of TNF‐α release in EOC20 cells. Interestingly, these findings are incongruent with earlier studies that indicate that Gαs‐mediated cAMP production can attenuate TNF‐α release from microglia and macrophages 43, 45. In light of this paradoxical evidence, it is plausible that α7 nAChR‐mediated intracellular calcium release can influence the effect of the cAMP pathway on the regulation of TNF‐α release. Indeed, calcium‐sensitive targets such as the cAMP‐responsive guanine nucleotide exchange factor (Epac) appear to play a key role in the inflammatory responses of microglia 57.

At this point, it is not possible to exclude the involvement of Gαq signaling in the anti‐inflammatory properties of α7 nAChRs in microglial cells as the results of the co‐IP experiment are inconclusive. In fact, various types of G proteins are likely capable of binding this receptor as suggested by proteomic findings from the rodent brain showing Gαi/s/q and various types of βγ within the α7 nAChR interaction network 17. Expression of α7_345‐8A_ in various types of cells shows that a mutation of the GPBC is sufficient to dramatically disrupt, but does not entirely inhibit, the interaction between G proteins and the α7 nAChR. This is underscored by the finding from this study that α7_345‐8A_ expression attenuates interaction between α7 nAChRs and Gαi, and earlier reports showing that α7_345‐8A_ expression virtually inhibits the α7 nAChR/Gαq interaction in PC12 cells 17. Additional M3‐M4 loop motifs outside of the GPBC may thus contribute to the interaction between the G protein and the receptor. In one scenario, the Gβγ complex, which has been shown to mediate both signaling and specificity for GPCRs 58, 59, may play an essential role in mediating the interaction between the nAChR and the G protein heterotrimer. The receptor‐associated Gβγ may also contribute to the activation of PLC by the α7 nAChR in our microglial cell line as shown previously in other cell types 56. In a second scenario, interaction between nAChRs and G proteins may be driven by intermediary actors such as scaffold proteins, which can bind both the receptor and the G protein. One such scaffold molecule is G protein‐regulated inducer of neurite outgrowth (Gprin) 1, which has been shown to mediate interaction between several receptors and G proteins in various types of cells 29, 60. Previously, we have shown that the interaction between α4 nAChRs and Gαi in T cells is directed by Gprin1 21. The existence of a similar signaling scaffold may direct the interaction between the α7 nAChR and specific G proteins in microglia.

## Author contributions

JRK and NK conceived and designed the project. JRK and TCG acquired the data. JRK and NK analyzed and interpreted the data. JRK and NK wrote the manuscript.
